# Heat Shock Factor 1 Is a Substrate for p38 Mitogen-Activated Protein Kinases

**DOI:** 10.1128/MCB.00292-16

**Published:** 2016-08-26

**Authors:** Sharadha Dayalan Naidu, Calum Sutherland, Ying Zhang, Ana Risco, Laureano de la Vega, Christopher J. Caunt, C. James Hastie, Douglas J. Lamont, Laura Torrente, Sudhir Chowdhry, Ivor J. Benjamin, Stephen M. Keyse, Ana Cuenda, Albena T. Dinkova-Kostova

**Affiliations:** a Division of Cancer Research, School of Medicine, University of Dundee, Dundee, Scotland, United Kingdom; b Division of Cardiovascular and Diabetes Medicine, School of Medicine, University of Dundee, Dundee, Scotland, United Kingdom; c Department of Immunology and Oncology, Centro Nacional de Biotecnología/CSIC, Madrid, Spain; d Department of Biology and Biochemistry, University of Bath, Claverton Down, Bath, United Kingdom; e Division of Signal Transduction Therapy, School of Life Sciences, University of Dundee, Dundee, Scotland, United Kingdom; f Division of Biological Chemistry and Drug Discovery, School of Life Sciences, University of Dundee, Dundee, Scotland, United Kingdom; g Cardiovascular Center, Medical College of Wisconsin, Milwaukee, Wisconsin, USA; h Department of Pharmacology and Molecular Sciences and Department of Medicine, Johns Hopkins University School of Medicine, Baltimore, Maryland, USA

## Abstract

Heat shock factor 1 (HSF1) monitors the structural integrity of the proteome. Phosphorylation at S326 is a hallmark for HSF1 activation, but the identity of the kinase(s) phosphorylating this site has remained elusive. We show here that the dietary agent phenethyl isothiocyanate (PEITC) inhibits heat shock protein 90 (Hsp90), the main negative regulator of HSF1; activates p38 mitogen-activated protein kinase (MAPK); and increases S326 phosphorylation, trimerization, and nuclear translocation of HSF1, and the transcription of a luciferase reporter, as well as the endogenous prototypic HSF1 target Hsp70. *In vitro*, all members of the p38 MAPK family rapidly and stoichiometrically catalyze the S326 phosphorylation. The use of stable knockdown cell lines and inhibitors indicated that among the p38 MAPKs, p38γ is the principal isoform responsible for the phosphorylation of HSF1 at S326 in cells. A protease-mass spectrometry approach confirmed S326 phosphorylation and unexpectedly revealed that p38 MAPK also catalyzes the phosphorylation of HSF1 at S303/307, previously known repressive posttranslational modifications. Thus, we have identified p38 MAPKs as highly efficient catalysts for the phosphorylation of HSF1. Furthermore, our findings suggest that the magnitude and persistence of activation of p38 MAPK are important determinants of the extent and duration of the heat shock response.

## INTRODUCTION

Heat shock factor 1 (HSF1) orchestrates an elaborate transcriptional program that enhances adaptation and survival under conditions of stress. It is activated in response to stresses such as heat shock, hypoxia, heavy metals, reactive oxygen species, and changes in pH. In an unstressed system, monomeric HSF1 is bound to its negative regulators, heat shock protein 40 (Hsp40), Hsp70, and Hsp90 (1–3). During stress, HSF1 is released from the complex and undergoes several activating posttranslational modifications that allow it to form a transcriptionally active trimer. In the nucleus, trimeric HSF1 binds to heat shock elements (HSE; comprising the consensus inverted repeat sequences nGAAn) to orchestrate the transcription of large networks of cytoprotective genes, including molecular chaperones, DNA damage repair components, and metabolic enzymes (4). Activation of HSF1 plays a vital role in human physiology and ageing, as well as in pathological processes such as cardiovascular disease, neurodegeneration, and cancer.

Increased nuclear HSF1 levels correlate with poor prognosis in breast, colon, and lung cancer (5, 6). Furthermore, it is becoming increasingly clear that HSF1 is able to support the malignant phenotype by orchestrating a transcriptional program beyond the heat shock response, including energy metabolism (5, 7). In addition, some of the downstream target genes of HSF1 encode proteins involved in global protein translation, such as the RNA-binding protein HuR (8, 9). Santagata et al. (10) have reported that the inhibition of protein translation in malignant cells reduced the activation of HSF1, providing an insight that a close relationship exists between the translational machinery and the transcriptional program orchestrated by HSF1. These findings raise the possibility of targeting HSF1 by inhibiting the cellular processes that lead to activation of the transcription factor in cancer.

The activity of HSF1 is controlled by a wide range of posttranslational modifications. Westerheide et al. (11) have reported that the activation of the deacetylase and longevity factor SIRT1 maintains HSF1 in a deacetylated, DNA-binding competent state and extends the duration of the heat shock response. Raychaudhuri et al. (12) discovered that EP300/CREB, a histone acetyltransferase, is responsible for stabilization of HSF1 through the acetylation of several of its lysine residues. In addition to acetylation, various phosphorylation modifications cause the transcription factor to become either transcriptionally repressed or activated. Most phosphorylation modifications occur within the regulatory domain (RD) of HSF1 and are inhibitory. Indeed, a recent study has shown that a phosphorylation-deficient HSF1 mutant, in which the 15 known phosphorylation sites within the RD had been disrupted (HSF1ΔpRD), is a potent transactivator under stress conditions and has a lower activation threshold than its wild-type (WT) counterpart (13). In human HSF1, phosphorylation at S303 (PP**S^303^**PPQ**S^307^**PRV) after obligatory priming phosphorylation at S307 by the mitogen-activated protein kinase (MAPK) extracellular signal-regulated kinase 1 (ERK1), is carried out by glycogen synthase kinase 3, inhibiting the function of the transcription factor (14–17). Similarly, phosphorylation at S121, catalyzed by MAPK-activated protein kinase 2 (MK2), inhibits the transcriptional activity of HSF1 and promotes its binding to Hsp90 (18). In stark contrast, phosphorylation at S326 (VDTLL**S^326^**PTAL) activates HSF1, and the mutation of S326 to alanine (S326A) reduces its transcriptional activity by >80% (19, 20). To our knowledge, the identity of the kinase(s) phosphorylating this site has not been clearly established.

**FIG 1:**
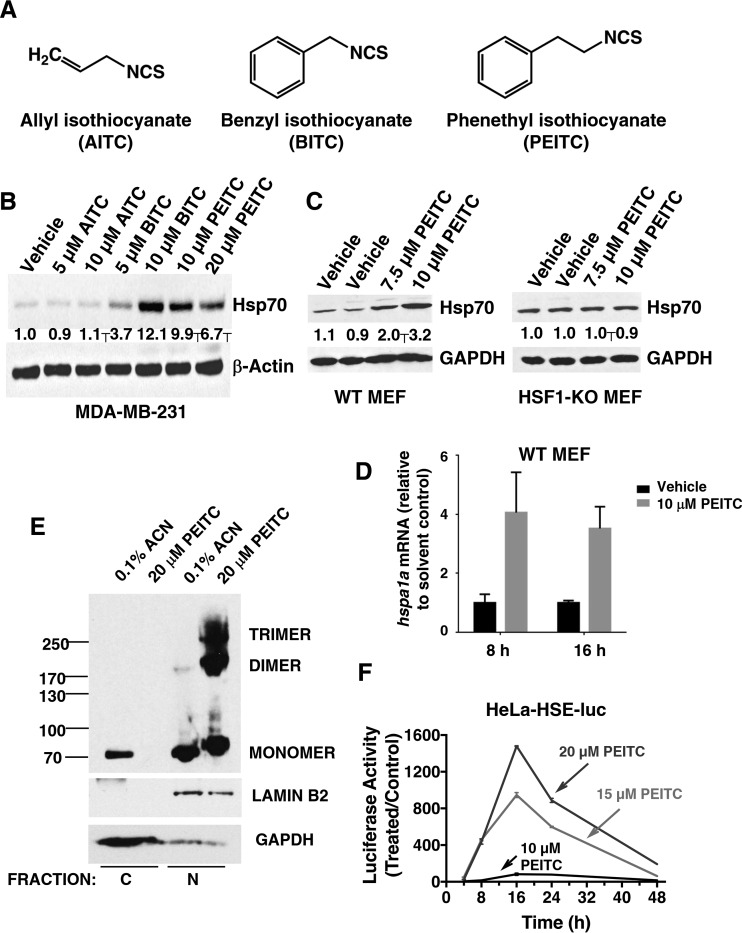
PEITC is a robust inducer of the heat shock response. (A) Chemical structures of allyl isothiocyanate (AITC), benzyl isothiocyanate (BITC), and phenethyl isothiocyanate (PEITC). (B and C) MDA-MB-231 cells (2.5 × 10^5^ per well) (B) or mouse embryonic fibroblasts (MEF, 2 × 10^5^ per well) (C) growing in six-well plates were exposed to vehicle (0.1% acetonitrile), AITC, BITC, or PEITC for 16 h. Cells were lysed in RIPA buffer, and proteins were resolved by SDS-PAGE, transferred to Immobilon-P membranes, and probed with an antibody against Hsp70. The levels of glyceraldehyde-3-phosphate dehydrogenase (GAPDH) served as loading control. (D) Wild-type MEF cells (2 × 10^5^ per well) in six-well plates were exposed to vehicle (0.1% acetonitrile) or PEITC for 8 or 16 h. The cells were lysed, and the total RNA was extracted and reverse transcribed into cDNA. The mRNA levels for *hspa1a* were quantified using real-time PCR. The data were normalized using β-actin as an internal control. (E) MDA-MB-231 cells (2 × 10^6^ per well) growing in 10-cm dishes were exposed to vehicle (0.1% acetonitrile) or 20 μM PEITC for 3 h. The cells were then fixed with 0.4% (wt/vol) paraformaldehyde. Cell lysates were subjected to nuclear (N) and cytoplasmic (C) separation, and proteins were resolved by SDS-PAGE (10% gel), transferred to Immobilon-P membranes, and probed with an antibody against HSF1. The levels of lamin B2 and GAPDH served as fraction purity indicators and as loading controls. (F) HeLa-HSE-luc cells (1.3 × 10^5^ per well) stably transfected with the luciferase gene under the control of the *HSP70.1* promoter were grown in 12-well plates and exposed to vehicle (0.1% acetonitrile) or PEITC. The luciferase activity was determined in cell lysates. The relative luminescence units (RLU) were quantified and normalized with respect to the vehicle control treatment. Data represent means ± the SD and are expressed as the ratio of the relative transcripts in treated to the control samples.

Diets rich in cruciferous vegetables have protective effects against neurodegenerative and cardiovascular disease, as well as cancer (21). Watercress (Nasturtium officinale), a vegetable from this family, is a rich source of the glucosinolate gluconasturtiin. Phenethyl isothiocyanate (PEITC) (Fig. 1A) is an isothiocyanate (ITC) that forms during plant tissue injury from gluconasturtiin, through the catalytic action of myrosinase (EC 3.2.1.147), a β-thioglucosidase (22, 23). PEITC is currently in clinical trials for the prevention of lung cancer and for the depletion of oral cells expressing mutant p53 in people who smoke (ClinicalTrials.gov). Due to the presence of the electrophilic isothiocyanate group, which reacts readily with sulfhydryl groups, PEITC is an activator of transcription factor nuclear factor-erythroid 2 p45-related factor 2 (NRF2), a master transcriptional regulator of antioxidant, anti-inflammatory, and drug-metabolizing enzymes. Global gene expression profiling of murine liver has revealed that, in addition to classical NRF2-dependent genes, a single dose (40 mg/kg) of orally administered PEITC induces transcription of heat shock proteins (24), but how this occurs is not known. Interestingly, PEITC has been reported to activate signal transduction cascades, including protein kinases (25). We show here that PEITC activates p38 MAPK, causes phosphorylation of HSF1 at S326, and transcriptionally activates HSF1. We further identify the family of p38 MAPK as highly efficient catalysts of HSF1 phosphorylation.

## MATERIALS AND METHODS

### Materials.

All general chemicals and reagents were of analytical grade and obtained from Sigma-Aldrich (Dorset, United Kingdom). PEITC was prepared as a stock solution in acetonitrile and diluted 1:1,000 in the cell culture medium before treatment. The concentration of the solvent was maintained at 0.1% (vol/vol) in all wells. The p38α/β MAPK inhibitor SB202190 was purchased from SYNkinase. The c-Jun N-terminal kinase (JNK) inhibitor JNK-In-8 was kindly provided by Dario Alessi (University of Dundee).

### Cell culture.

MDA-MB-231 cells were from ATCC. HeLa-HSE-luc cells (26) were a generous gift from Richard I. Morimoto (Northwestern University, USA). Mouse embryonic fibroblasts (MEFs) from wild-type or HSF1-knockout mice were isolated as described previously (27). The human epidermoid cancer cell line A431 and the production and transduction of lentivirus short hairpin RNA to generate stable clones, which do not express p38γ or p38δ, have been described (28). All cell lines were maintained at 5% CO_2_ in air at 37°C and were cultured in Dulbecco modified Eagle medium (DMEM) supplemented with 10% (vol/vol) heat-inactivated fetal bovine serum. The medium in which HeLa-HSE-luc cells were grown also contained 100 μg/ml G418 (Invitrogen), whereas the medium for MEF cells was additionally supplemented with nonessential amino acids and 50 U/ml penicillin-streptomycin.

### Western blotting.

Cells grown in six-well plates were washed twice with phosphate-buffered saline (PBS) and lysed in 150 μl of either radioimmunoprecipitation assay (RIPA) buffer (50 mM Tris-Cl [pH 7.5], 150 mM NaCl, 0.5% [wt/vol] sodium deoxycholate, 1% NP-40 [vol/vol], 0.1% SDS [wt/vol], and 1 mM EDTA containing 1 protease inhibitor cocktail tablet [Roche] per 10 ml of buffer) or SDS lysis buffer (50 mM Tris-Cl [pH 6.8], 2% [wt/vol] SDS, 10% [vol/vol] glycerol, and 0.005% bromophenol blue). The lysates derived from RIPA buffer were transferred into 1.5-ml Eppendorf tubes, which were placed on a rotator at 4°C for 30 min. The cell debris was then removed by centrifugation at 16,300 × *g* for 10 min at 4°C, and the supernatant was transferred to a new tube. The lysates derived from the SDS lysis buffer were subjected to sonication at 20% amplitude for 20 s. A BCA assay (Thermo) was used to determine protein concentrations. Proteins were resolved by SDS-PAGE, transferred to Immobilon-P membranes, and probed with specific antibodies against Hsp70 (mouse monoclonal, 1:1,000; StressMarq, York, United Kingdom), Hsp90 (mouse monoclonal, 1:5,000; BD Biosciences, New Jersey), HER2 (rabbit polyclonal, 1:500; Millipore, CA), RAF1 (rabbit polyclonal, 1:200; Santa Cruz, CA), HSF1 (rabbit polyclonal, 1:1,000; Enzo Life Sciences, Exeter, United Kingdom), pS326-HSF1 (rabbit polyclonal, 1:10,000; Abcam, Cambridge, United Kingdom), p38 MAPK (rabbit polyclonal, 1:1,000; Cell Signaling, MA), pp38 MAPK (rabbit polyclonal, 1:1,000; Cell Signaling), JNK1/2 (rabbit polyclonal, 1:1,000; Cell Signaling), pJNK1/2 (rabbit polyclonal, 1:1,000; Biosource Europe, Nivelles, Belgium), pERK1/2 (rabbit polyclonal, 1:1,000; Cell Signaling), pT334-MK2 (rabbit polyclonal, 1:1,000, Cell Signaling), and pS235/6 S6 (rabbit polyclonal, 1:5,000l Cell Signaling). Isoform-specific p38γ and p38δ MAPK antibodies were from the Division of Signal Transduction Therapy and were used at a concentration of 1 μg/ml. Equal loading was confirmed by probing the blots with antibodies against GAPDH (glyceraldehyde-3-phosphate dehydrogenase; rabbit polyclonal, 1:5,000) or β-actin (mouse monoclonal, 1:10,000), both from Sigma (Dorset, United Kingdom) or lamin A (rabbit polyclonal, 1:1,000; GeneTex, Irvine, CA). The Western blots shown are representative of at least three independent experiments.

### Nuclear-cytoplasmic separation.

MDA-MB-231 cells (10^6^ per dish) were plated in 6-cm dishes and treated for the indicated periods of time with 0.1% (vol/vol) acetonitrile or PEITC. The REAP method described by Suzuki et al. (29) was used to obtain separate cytoplasmic and nuclear fractions. In short, cells were washed twice with ice-cold PBS (pH 7.5), collected in 500 μl of ice-cold PBS, transferred to Eppendorf tubes, and subjected to centrifugation at 10,000 × *g* for 30 s at room temperature. Next, the supernatant was discarded, and the pellet was resuspended in 450 μl of ice-cold 0.1% NP-40 (vol/vol) in PBS. The lysates were then subjected to a further centrifugation at 10,000 × *g* for 30 s at room temperature. The supernatant was collected as the cytoplasmic fraction. One volume of 5× sample SDS loading buffer (250 mM Tris-Cl [pH 6.8], 10% [vol/vol] SDS, 50% (vol/vol) glycerol, and 0.025% [wt/vol] bromophenol blue) was added to 4 volumes of the cytoplasmic fraction, and the samples were heated for 5 min at 100°C and subjected to SDS-PAGE. The remaining pellet containing the nuclear fraction was washed twice with ice-cold 0.1% NP-40 (vol/vol) in PBS and dissolved in 1× sample loading buffer (50 mM Tris-Cl [pH 6.8], 2% [vol/vol] SDS, 10% [vol/vol] glycerol, and 0.005% [wt/vol] bromophenol blue) and heated for 5 min at 100°C. The nuclear fractions were sonicated before subjecting them to SDS-PAGE.

### Quantitative real-time PCR.

The primers and probes for quantifying the levels of the mRNA species were from Applied Biosystems (*hspa1a*, Mm01159846_s1; *HER2*, HS01001580_m1; and *RAF1*, HS00234119_m1). Cells (2 × 10^5^ per well) were seeded in six-well plates. After 24 h, the cells were exposed to vehicle (0.1% acetonitrile) or PEITC for a further 8 h (MEFs) or 16 h (MEFs and MDA-MB-231 cells). After cell lysis, total RNA was extracted using RNeasy kit (Qiagen, Ltd.), and 500 ng of total RNA was reverse transcribed into cDNA with an Omniscript reverse transcription kit (Qiagen, Ltd.). Real-time PCR was performed on an Applied Biosystems 7900HT Fast real-time PCR system. The data were normalized using β-actin (mouse ACTB [Applied Biosystems], Mm00607939_s1) as an internal control.

### Luciferase assay.

HeLa-HSE-luc cells (10^5^ per well) were seeded in each well of a 24-well plate and 24 h later treated with PEITC or 0.1% (vol/vol) acetonitrile vehicle for 8, 16, 24, or 48 h. The cells were washed twice with 0.1% PBS, and 100 μl of 1× reporter lysis buffer (Promega) was added to each well. The plate was placed at −20°C for a minimum for 2 h and then transferred to thaw on a shaker at room temperature for 30 min. Cell lysates were collected into Eppendorf tubes and subjected to centrifugation at 15,000 × *g* for 2 min at 4°C. The luciferase activity was measured in 10 μl of cell lysate in opaque 96-well plates (Corning) using a microplate-reader based luminometer (Orion II; Berthold) and normalized for protein concentration determined by a Bradford assay (Bio-Rad).

### ATP-binding assay.

MDA-MB-231 cells (0.5 × 10^6^ per dish) were seeded in 6-cm dishes. After 24 h, the cells were treated for a further 24 h with 0.1% acetonitrile as the vehicle control for sulfoxythiocarbamate alkyne (STCA; 75 μM) and PEITC (20 μM) treatments or with 0.1% dimethyl sulfoxide (DMSO) as the vehicle control for the geldanamycin (GA; 1 μM) and celastrol (CL; 0.8 μM) treatments. The cells were harvested by scraping into 300 μl of lysis buffer (10 mM Tris [pH 7.5], 150 mM NaCl, and 0.25% NP-40, with one protease inhibitor tablet [Roche] per 10.0 ml of buffer), frozen, thawed, and lysed for 30 min at 4°C. ATP-agarose beads (Jena Bioscience) were washed with the incubation buffer (10 mM Tris [pH 7.5], 150 mM NaCl, 20 mM MgCl_2_, 0.05% NP-40, and 1 mM DTT). Cell lysates (200 μg of total protein) were added to a suspension of 30 μl of beads in 1.25 ml of buffer, and the samples were incubated with rotation overnight at 4°C. The beads were collected by centrifugation and washed three times with the incubation buffer. SDS loading buffer (10 μl) and incubation buffer (40 μl) were added to the beads, and the samples were incubated for 5 min at 100°C. The beads were pelleted by centrifugation, and the supernatants were collected and subjected to Western blot analysis.

### Detection of HSF1 trimerization.

MDA-MB-231 (2 × 10^6^ per dish) cells were grown on 10-cm dishes for 24 h and treated with 0.1% acetonitrile or 20 μM PEITC for a further 3 h. The cells were then washed twice with PBS. Next, 10 ml of 0.4% (wt/vol) paraformaldehyde in PBS (0.4% PFA-PBS) was added to the dishes over 10 min, where fresh 0.4% PFA-PBS was added every 5 min. Next, the PFA-PBS was removed, and the reaction was quenched with the addition of 3 ml of ice-cold 1.25 M Glycine-PBS. After washing twice with PBS, nuclear and cytoplasmic fractions were obtained. The cells were lysed in buffer A (10 mM KCl, 5 mM MgCl_2_, 50 mM Tris-Cl [pH 7.5], 0.5% [vol/vol] NP-40, 1 mM DTT, and one EDTA-free complete mini-protease inhibitor cocktail tablet [Roche] and one phos-STOP tablet [Roche] per 10 ml of buffer). The lysates were subjected to centrifugation at 1,000 × *g* for 5 min at 4°C. The supernatant containing the cytoplasmic fraction was transferred to a fresh Eppendorf tube where one volume of 5× SDS sample loading buffer was added to four volumes of the cytoplasmic fraction. The pellet containing the nuclear fraction was washed there times with the buffer A before dissolving it in 1× sample loading buffer (50 mM Tris-Cl [pH 6.8], 2% [wt/vol] SDS, 10% [vol/vol] glycerol, and 0.005% [wt/vol] bromophenol blue). The nuclear fractions were subjected to sonication. Both the cytoplasmic and the nuclear fractions were subjected to SDS-PAGE before immunoblotting.

### Coimmunoprecipitation.

MDA-MB-231 (4 × 10^6^ per dish) cells were grown on 10-cm dishes for 24 h and then treated with 0.1% DMSO or 20 μM PEITC for 45 min. The dishes with cells were placed on ice and washed twice with ice-cold PBS. Protein G-Dynabeads (30 μl slurry [Invitrogen]) were washed twice for 5 min with PBS and incubated with 1 μg of mouse monoclonal HSF1 antibody (Santa Cruz) for 1 h at room temperature, after which the beads were washed three times every 10 min with PBS. Cells were lysed with 1.0 ml ice-cold CO-IP buffer (150 mM NaCl, 50 mM Tris-Cl [pH 7.4], 1 mM EDTA, 1% NP-40, 0.1% [wt/vol] sodium deoxycholate) supplemented with one EDTA-free protease cocktail inhibitor tablet (Roche) and one phosphatase inhibitor tablet (PhoSTOP Roche). The cell lysates were passed through a 23-gauge needle 10 times before they were clarified by centrifugation at 4°C for 30 min at 16,000 × *g*. Then, 50 μl of the clarified lysate (IP sample) was transferred to a fresh Eppendorf tube to serve as an input sample. To preclear the IP sample, 30 μl of protein G-Dynabead slurry was washed twice for 5 min with PBS, and the beads were added to each of the IP sample (containing 0.8 to 1.0 mg of protein) and then incubated for 1 h at 4°C on a tube rotator. Subsequently, the protein G-Dynabead-antibody conjugate was added to the precleared IP sample, followed by incubation for 16 h at 4°C on a tube rotator. The immunoprecipitated complexes were washed three times with ice-cold CO-IP buffer every 10 min and then eluted from the beads by adding 70 μl of 1× LDS buffer (Invitrogen) and heating the sample at 70°C for 10 min. After cooling, 7 μl of sample reducing agent (Invitrogen) was added to the sample, followed by incubation for 15 min at room temperature. Immunoprecipitated proteins (35 μl) were resolved by electrophoresis. Antibodies against Hsp90 (monoclonal; BD Biosciences) and HSF1 (rabbit polyclonal; Enzo Life Sciences) were used for detection of the respective proteins.

### Generation of p38γ and p38δ stable knockdown cell lines.

p38γ and p38δ expression was reduced by RNA interference using Mission shRNA constructs (Sigma; plasmid clone IDs TRCN0000006145 and TRCN0000006147 for p38γ and plasmid clone IDs TRCN0000000827 and TRCN0000009979 for p38δ). A lentivirus containing the control pLKO.1 or the shRNA plasmids was used to infect MDA-MB-231 cells. To produce the virus, HEK293T cells were transfected using Lipofectamine 2000 (Invitrogen) with empty pLKO.1-puro vector or the shRNA constructs against p38γ or p38δ, together with the packaging vectors (psPAX2 and pMD2.G) in serum-reduced medium. On the following day, the medium was replaced with complete DMEM and, after 24 h, the lentivirus-containing supernatant was collected, filtered, and used to transduce MDA-MB-231 cells. Cells containing the shRNA plasmid were selected, expanded, and maintained with supplementation of puromycin (2 mg/ml) for approximately 3 weeks, during which time cell lysates were collected every 3 to 4 days to ensure the respective p38 expression levels were reduced throughout the selection period.

### Expression and purification of recombinant hexahistidine-tagged HSF1.

Full-length HSF1 cDNA was amplified by PCR from a plasmid obtained from Addgene (plasmid ID 32537, in which the cDNA sequence was found to have a nucleotide substitution at position 1343 from a C to a T, leading to a change from P to L at position 448 in the protein sequence) and subcloned into the bacterial expression vector pet15b using NdeI and XhoI. After transformation into E. coli [BL21(DE3)pLysS], the cells were grown to an optical density at 600 nm of 0.6, and induced for a further 3.5 h at 37°C with 400 μM IPTG (isopropyl-β-d-thiogalactopyranoside). The induced cells were harvested by centrifugation and resuspended in extraction buffer (20 mM Tris-Cl [pH 7.9], 150 mM NaCl, 5 mM imidazole, and 0.01% [vol/vol] IGEPAL CA-630). After freezing and thawing, the cells were disrupted by sonication for 5 min on ice. Cell debris were then cleared by centrifugation at 10,000 × *g* for 15 min at 4°C. The resultant supernatant was left on ice for 30 min before it was applied to nickel agarose resin (His-TrapHP; GE Healthcare). The resin was washed with 20 mM Tris-Cl (pH 7.9), 150 mM NaCl, and 5 mM imidazole. The supernatant (20 ml) was then incubated for 1 h at 4°C with 1 ml of resin. After three washes with buffer, the protein was eluted with 2 ml of 20 mM Tris-Cl (pH 7.9), 150 mM NaCl, and 250 mM imidazole. To remove the imidazole, the preparation was dialyzed in 50 mM Tris-Cl (pH 7.4)–150 mM NaCl. Mutant S326A HSF1 was generated by site-directed mutagenesis of the plasmid vector pet15b containing the HSF1 cDNA by using the primers 5′-GTGGACACCCTCTTGGCCCCGACCGCCCTCATTG-3′ and 5′-CAATGAGGGCGGTCGGGGCCAAGAGGGTGTCCAC-3′ and a QuikChange II mutagenesis kit (Stratagene). The hexahistidine-tagged mutant S326A HSF1 recombinant protein was generated using the method described above.

### High-content microscopy and analysis.

HSF1-knockout MEFs were seeded in black-walled 96-well plates (Corning Costar 3904) at 4 × 10^3^ cells/well and transfected with 50 ng/well green fluorescent protein (GFP)-tagged wild-type or S326A or S326E mutants of HSF1 using Lipofectamine LTX reagent (Invitrogen), using eight replicate wells per condition. At 24 h after transfection, the cells were fixed using 4% paraformaldehyde in PBS for 10 min at room temperature and permeabilized using methanol at −20°C for 5 min. The cells were blocked using 2.5% normal goat serum in PBS–0.1% sodium azide and counterstained using rabbit anti-ERK1/2 monoclonal antibody (clone 137F5; Cell Signaling Technology) and Alexa Fluor 546-labeled highly cross-adsorbed goat anti-rabbit secondary antibody (Invitrogen). DNA was labeled with 300 nM DAPI (Sigma) in PBS, and the images were acquired using an IN Cell Analyzer 2000 robotic fluorescence microscope (GE Healthcare) using a 20× lens to capture four fields per well for each fluorophore (DAPI, GFP, and Alexa Fluor 546) using 2×2 pixel binning to maximize the signal/noise ratio. The images were analyzed using a custom algorithm constructed within IN Cell Developer software (GE Healthcare), using DAPI and ERK1/2 images to identify nuclear and cytoplasmic regions, respectively, in order to assess fluorescence distribution within the GFP channel.

### Kinase assays.

The incubation mixtures contained purified recombinant kinase (at a specific activity of either 6 or 0.06 mU/μl), recombinant HSF1 (1 μg) substrate, 10 mM MgCl_2_, 0.1 mM [γ-^32^P]ATP (approximately 0.5 × 10^6^ cpm/nmol), and kinase buffer (50 mM Tris-Cl [pH 7.4], 0.03% [vol/vol] Brij-35, 0.1% [vol/vol] β-mercaptoethanol) in a total volume of 50 μl. The kinase assays were performed at 30°C. At the times indicated, a 15-μl aliquot of each incubation mixture was removed, the reaction was terminated by the addition of SDS gel loading buffer, the sample was loaded on SDS-PAGE, and the excess [γ-^32^P]ATP was removed by electrophoresis. The gels were dried and subjected to autoradiography. Protein-containing gel pieces (visualized by staining with Coomassie brilliant blue) were then excised, and phosphate incorporation into HSF1 was quantified by scintillation counting.

Cold assays were performed in an analogous manner using purified recombinant kinase (at a specific activity of 0.06 mU/μl), recombinant HSF1 (1 μg) substrate, MgCl_2_ (10 mM), and ATP (0.1 mM) instead of [γ-^32^P]ATP. For identification of the phosphorylated sites, the gel bands were excised, reduced with DTT (10 mM), alkylated with iodoacetamide (50 mM), and digested overnight (16 h) with trypsin (modified sequencing grade; Roche) at 30°C. The resulting peptides were extracted from the gel, dried in a SpeedVac concentrator (Thermo Scientific), resuspended in 10 μl of 5% formic acid, and diluted five times. Any residual particles were removed by centrifugation, the samples were then transferred to high-pressure liquid chromatography vials, and analyzed by liquid chromatography-tandem mass spectrometry on an Ultimate3000 RSLCnano System (Thermo Scientific) coupled to a LTQ OrbiTrap VelosPro (Thermo Scientific) with an EasySpray source. The data files were analyzed with Proteome Discoverer (v1.4.1) using Mascot (v2.4.1) as the search engine using a protein-specific database (HisTag-HSF1) and the IPI-Human (ipi.HUMAN.v3.87) database.

### Statistical analysis.

Values are expressed as ±1 standard deviation (SD). The differences between groups were determined by using a Student *t* test. Analyses were performed using Excel (Microsoft Corp.).

## RESULTS

### Cysteine-reactive PEITC induces the heat shock response.

We have previously reported that structurally diverse NRF2 activators, all of which react with sulfhydryl groups, induce the heat shock response, and demonstrated the essential requirement for HSF1 (30). The isothiocyanates represent a prominent class of NRF2 activators, which have shown chemoprotective effects in numerous animal models of chronic disease; some have been and/or currently are in clinical trials (31–33). We therefore examined the potential heat shock response-inducer activity of three representative isothiocyanates: allyl (AITC)-, benzyl (BITC)-, and phenethyl (PEITC) isothiocyanates (Fig. 1A) in the human breast cancer cell line MDA-MB-231, using Hsp70 as a prototypic heat shock protein. When cells were exposed for 24 h to the aromatic isothiocyanates BITC or PEITC at a concentration of 10 μM, the levels of Hsp70 increased by ∼12- and ∼10-fold, respectively, whereas the levels of Hsp70 remained unchanged upon exposure to 10 μM the aliphatic isothiocyanate AITC (Fig. 1B). PEITC is in clinical trials for prevention of lung cancer and for depletion of oral cells expressing mutant p53 (ClinicalTrials.gov). We therefore focused our subsequent studies on this isothiocyanate. Experiments in MEFs confirmed the requirement for HSF1 for the induction of Hsp70 by PEITC. In wild-type MEFs, exposure to 7.5 or 10 μM PEITC for 24 h caused upregulations of Hsp70 by ∼2- and ∼3.2-fold, respectively, whereas the levels of this heat shock protein remained unchanged in their HSF1-deficient counterparts (Fig. 1C). Consistent with the increase in the protein levels of Hsp70, the mRNA levels for *hspa1a* were upregulated by 4.1- and 3.5-fold after the exposure of wild-type MEF cells to 10 μM PEITC for 8 or 16 h, respectively (Fig. 1D).

Nuclear-cytoplasmic separation experiments conducted in MDA-MB-231 cells showed that PEITC caused nuclear translocation of HSF1 (Fig. 1E). Thus, in vehicle-treated cells, HSF1 was present in both the cytoplasmic and the nuclear fractions. In sharp contrast, in the cytoplasmic fraction of cells treated with PEITC for 3 h, there was no detectable HSF1, and essentially all of the HSF1 was in the nuclear fraction. Furthermore, the presence of monomeric, dimeric, and trimeric HSF1 species was readily detectable in the nuclear fractions of PEITC-treated cells. Collectively, these experiments show that, upon PEITC treatment, HSF1 undergoes nuclear translocation and trimerization. Notably, the gel electrophoretic mobility of monomeric HSF1 in the nuclear fractions of PEITC-treated cells was slower than in their vehicle-treated counterparts (Fig. 1E), indicating occurrence of posttranslational modifications.

Trimerization is required for the transcriptional activity of HSF1 (34–37). To test whether the HSF1 trimers that form upon treatment with PEITC are able to enhance transcription through heat shock elements (HSEs), we used the cervical cancer HeLa HSE-luciferase reporter cell line (HeLa-HSE-luc) stably transfected with the *HSP70.1* promoter fused to the luciferase gene (26). Remarkably, PEITC led to a dramatic dose- and time-dependent induction of the reporter, with a maximal increase of more than 1,000-fold (Fig. 1F). Together, these results demonstrate that PEITC is a potent and robust inducer of the heat shock response.

### PEITC inhibits Hsp90.

**FIG 2:**
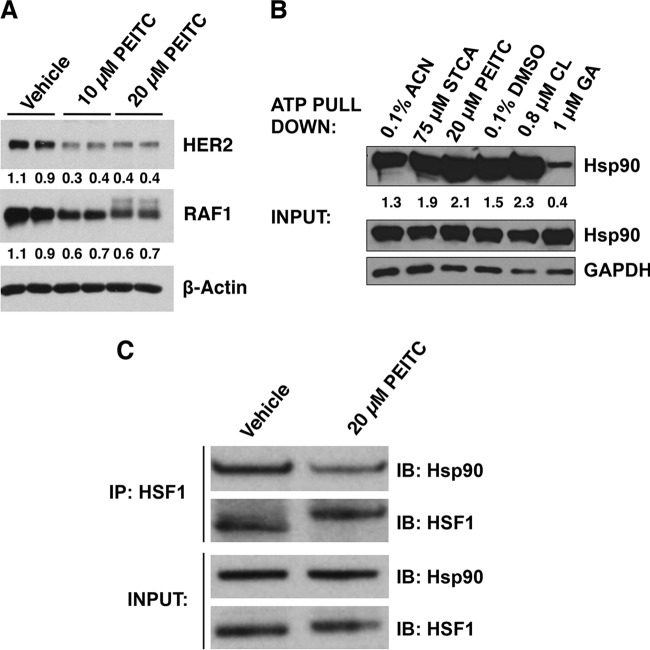
PEITC inhibits Hsp90. (A) MDA-MB-231 cells (2.5 × 10^5^ per well) in six-well plates were treated with vehicle (0.1% acetonitrile) or PEITC for 24 h. The levels of HER2 and RAF1 were detected by Western blot analysis. The levels of β-actin served as a loading control. (B) MDA-MB-231 cells (0.5 × 10^6^ per dish) were grown in 6-cm dishes. After 24 h, the cells were treated for a further 24 h with 0.1% acetonitrile (ACN) as the vehicle control for sulfoxythiocarbamate alkyne (STCA) and PEITC treatments or with 0.1% DMSO as the vehicle control for the geldanamycin (GA) and celastrol (CL) treatments. The cells were lysed and subjected to ATP pulldown using ATP-agarose beads. For the ATP pulldown and input samples, Hsp90 or GAPDH were detected by Western blot analyses. (C) MDA-MB-231 cells (2.5 × 10^5^ per well) were grown in six-well plates and treated with vehicle (0.1% acetonitrile) or PEITC for 45 min. Cells were lysed and subjected to immunoprecipitation with an anti-HSF1 antibody and then immunoblotted with an anti-Hsp90 antibody. An aliquot of total lysate was subjected to immunoblot analysis with anti-Hsp90 and anti-HSF1 antibodies.

Activation of HSF1 requires release from its negative regulators; indeed, inhibition of Hsp90, the main negative regulator of HSF1, often leads to induction of the heat shock response (38). To test whether PEITC inhibits the function of Hsp90, we evaluated the stability of two well-established Hsp90 client oncoproteins, the tyrosine kinase HER2 and the serine/threonine kinase RAF1, both of which bind strongly to Hsp90 (39). After treatment with PEITC, the levels of HER2 and RAF1 were decreased by ∼60 and ∼35%, respectively, a finding consistent with Hsp90 inhibition (Fig. 2A). Treatment with 10 μM PEITC did not lead to any changes in the transcription of either of these genes, as quantified by real-time PCR. Interestingly, however, the 20 μM PEITC treatment led to a significant (*P* = 0.002, *n* = 3) 45% decrease in the mRNA levels for HER2, although the mRNA levels for RAF1 were not affected (not shown). The reason for this decrease in HER2 expression and its potential contribution to the decreased HER2 protein levels at the high concentrations of PEITC are presently unknown. In contrast to geldanamycin (GA), an Hsp90 inhibitor that competes with ATP, but similarly to celastrol (CL) and sulfoxythiocarbamate alkyne (STCA), which inhibit Hsp90 by modifying its cysteine residues without interfering with ATP binding (40), PEITC did not prevent the ability of the chaperone to bind ATP (Fig. 2B). These results support the notion that PEITC, by virtue of its cysteine reactivity inhibits Hsp90 and further suggest that, by inhibiting Hsp90, the isothiocyanate may trigger the release of HSF1. Indeed, immunoprecipitation experiments showed that the amount of HSF1 bound to Hsp90 is greatly reduced upon exposure to PEITC (Fig. 2C).

### PEITC causes phosphorylation of HSF1 at S326.

**FIG 3:**
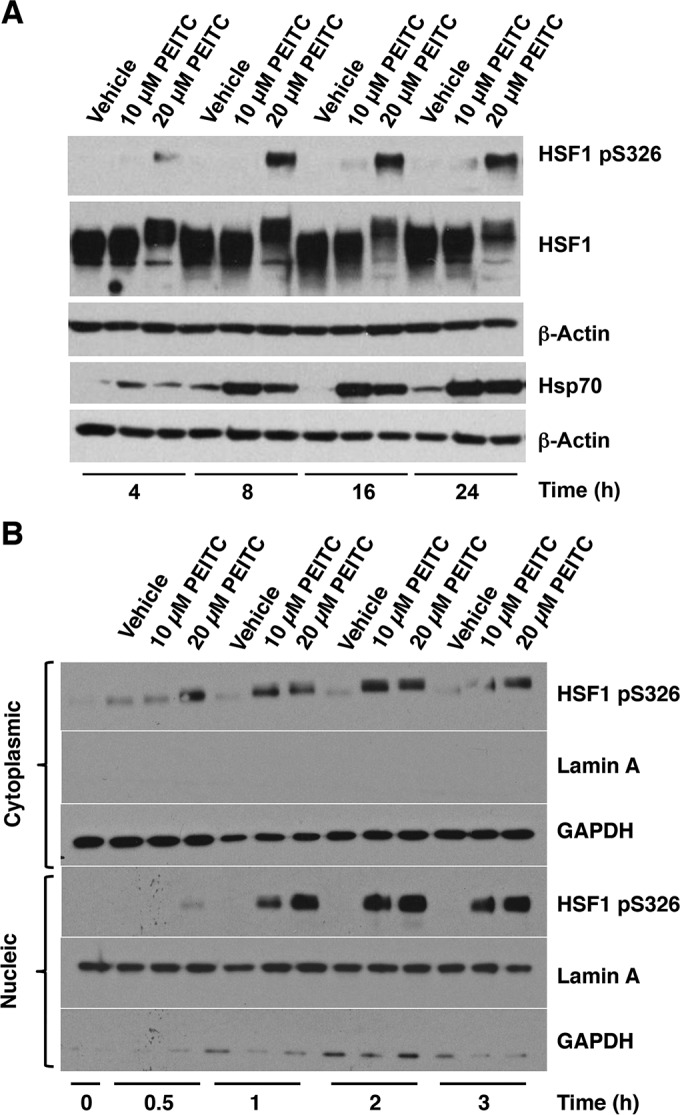
PEITC causes phosphorylation of HSF1 at S326. MDA-MB-231 cells (2.5 × 10^5^ per well) in six-well plates were treated with vehicle (0.1% acetonitrile) or PEITC for either 24 h (A) or for the indicated periods of time (B). In panel A, the levels of pS326 HSF1, total HSF1, and Hsp70 were detected by Western blot analysis in cell lysates, and the levels of β-actin served as loading control. In panel B, the levels of pS326 HSF1 were detected by Western blot analysis in the cytoplasm and nuclei after nuclear-cytoplasmic separation. The levels of lamin A and GAPDH served as fraction purity indicators and as loading controls.

In addition to release from Hsp90, full transcriptional activation of HSF1 requires its phosphorylation at S326 (19, 20). The shift in gel electrophoretic mobility of the monomeric form of HSF1 in nuclear fractions of PEITC-treated cells (Fig. 1E) indicated the occurrence of posttranslational modifications. Given the dramatic activation of the HSE-luciferase reporter by PEITC (Fig. 1F), we tested the possibility that exposure to PEITC was causing HSF1 phosphorylation at S326. The use of a pS326-phosphospecific antibody revealed a time- and dose-dependent increase in pS326 HSF1 and an upregulation of Hsp70 upon exposure to PEITC (Fig. 3A). Notably and in full agreement with the shift in electrophoretic mobility of HSF1 in nuclear fractions of PEITC-treated cells (Fig. 1E), as well as in the immunoprecipitation experiment (Fig. 2C), in this experiment the migration of HSF1 was slower in lysates from cells treated with 20 μM PEITC than from vehicle- or 10 μM PEITC-treated cells (Fig. 3A). Surprisingly, however, although HSF1 S326 phosphorylation was more extensive upon treatment with the higher concentration (20 μM) of PEITC, induction of Hsp70 appeared to be greater at the lower concentration (10 μM) of the isothiocyanate. Nuclear-cytoplasmic separation further confirmed the accumulation of pS326 HSF1 in both cytoplasmic and nuclear fractions (Fig. 3B). The results from this shorter time course experiment also indicated that HSF1 phosphorylation occurred in the cytoplasm and preceded the nuclear translocation of the transcription factor.

### PEITC activates p38 MAPK.

**FIG 4:**
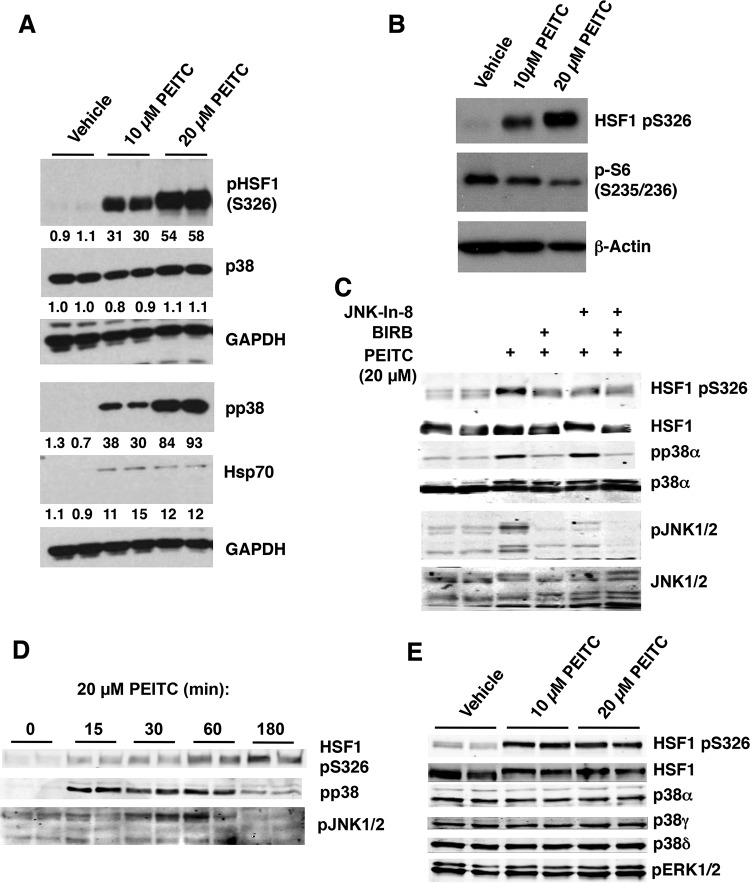
PEITC activates p38 and JNK1/2 MAPK, and inhibits mTOR. MDA-MB-231 cells (2.5 × 10^5^ per well) growing in six-well plates were treated with vehicle (0.1% acetonitrile) or PEITC for either 24 h (A and B), 3 h (C and E), or for the indicated periods of time (D). The levels of HSF1, pS326 HSF1, pS235/6 S6, Hsp70, the p38 isoforms α, γ, and δ, phosphorylated p38 (pp38), phosphorylated p38α (pp38α), JNK1/2, and phosphorylated JNK1/2 (JNK1/2) were detected by Western blot analysis.

S326 of HSF1 represents a proline-directed phosphorylation site. Only a stringent subset of kinases, known as CMGC kinases are able to phosphorylate proline-directed sites (41). Since PEITC has been reported to activate p38 mitogen-activated protein kinases (MAPKs) (25), a proline-directed family of kinases, we next examined their status in MDA-MB-231 cells. A dose-dependent phosphorylation of p38 MAPK was readily detectable and increased by ∼30- and ∼90-fold after treatment with 10 and 20 μM PEITC, respectively (Fig. 4A). Importantly, the levels of p38 MAPK were unchanged, showing that PEITC did not cause any alterations in protein expression or stability of the kinases. In contrast to the activation of p38 MAPK, exposure to PEITC decreased the phosphorylation of the ribosomal subunit S6 at S235/236 (Fig. 4B), indicating inhibition of the mechanistic target of rapamycin (mTOR), a kinase that had been previously implicated in the phosphorylation of HSF1 at S326 (20). Moreover, the dose-dependent PEITC-mediated phosphorylation of p38 MAPK correlates well with the extent of phosphorylation of HSF1 at S326, which increased by 30- and 55-fold after treatment with 10- and 20 μM PEITC, respectively (Fig. 4A).

It has been reported that the c-Jun N-terminal kinases (JNK) phosphorylate and activate HSF1 (42). We therefore next tested the effect of JNK-In-8, a JNK-selective inhibitor (43), and BIRB0796, a p38 MAPK inhibitor (44), on the ability of PEITC to induce phosphorylation of HSF1 at S326. Both inhibitors decreased the PEITC-mediated phosphorylation of HSF1 at S326 (Fig. 4C). However, a time course experiment further revealed that, although both p38 and JNK1/2 were activated by exposure to 20 μM PEITC, the activation of JNK1/2 displayed a delayed kinetics in comparison to the kinetics of activation of p38 MAPK (Fig. 4D).

**FIG 5:**
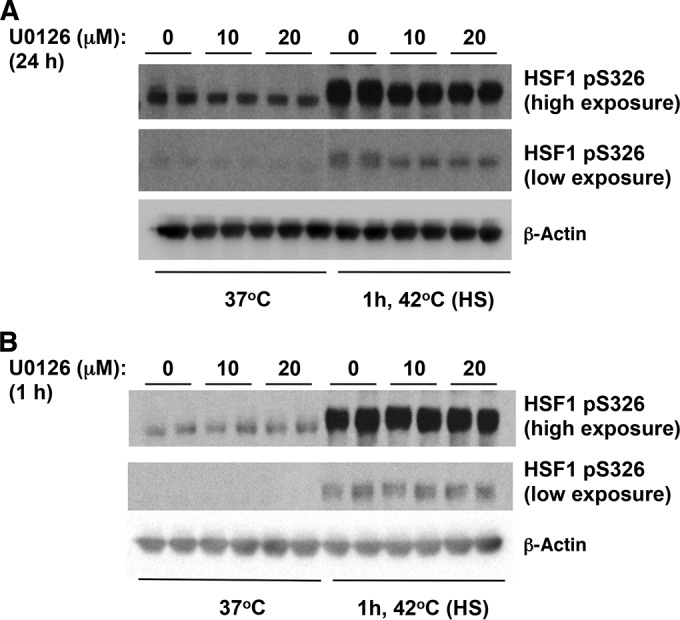
The MEK inhibitor U0126 reduced the levels of heat shock induced phosphorylation of HSF1 at S326 after a 24-h pretreatment, but a 1-h pretreatment had no effect. MDA-MB-231 cells (2.5 × 10^5^ per well) in six-well plates were pretreated with U0126 for 24 h (A) or 1 h (B) and subsequently subjected to heat shock (HS). The levels of HSF1 and pS326 HSF1 were detected by Western blot analysis. The levels of β-actin served as a loading control.

Recently, it was reported that HSF1 physically interacts and is phosphorylated at S326 by MAPKK (also known as MEK) (45). We therefore next examined the effect of inhibiting MEK on HSF1 S326 phosphorylation using the highly selective MEK1/2 inhibitor 1,4-diamino-2,3-dicyano-1, 4-bis[2-aminophenylthio]butadiene (U0126) (46). In agreement with the published report (45), we were able to detect reduced levels of phosphorylation of HSF1 at S326 in lysates of heat-shocked cells that had been pretreated for 24 h with U0126 (Fig. 5A). However, the MEK inhibitor had no effect on the phosphorylation of HSF1 at S326 in heat-shocked cells that had been pretreated with U0126 for 1 h (Fig. 5B). Overall, these findings raise the possibility that p38 MAPK represents one group of the long-sought catalysts for the phosphorylation of this key residue.

### p38 MAPK phosphorylate HSF1 at S326 *in vitro*.

**FIG 6:**
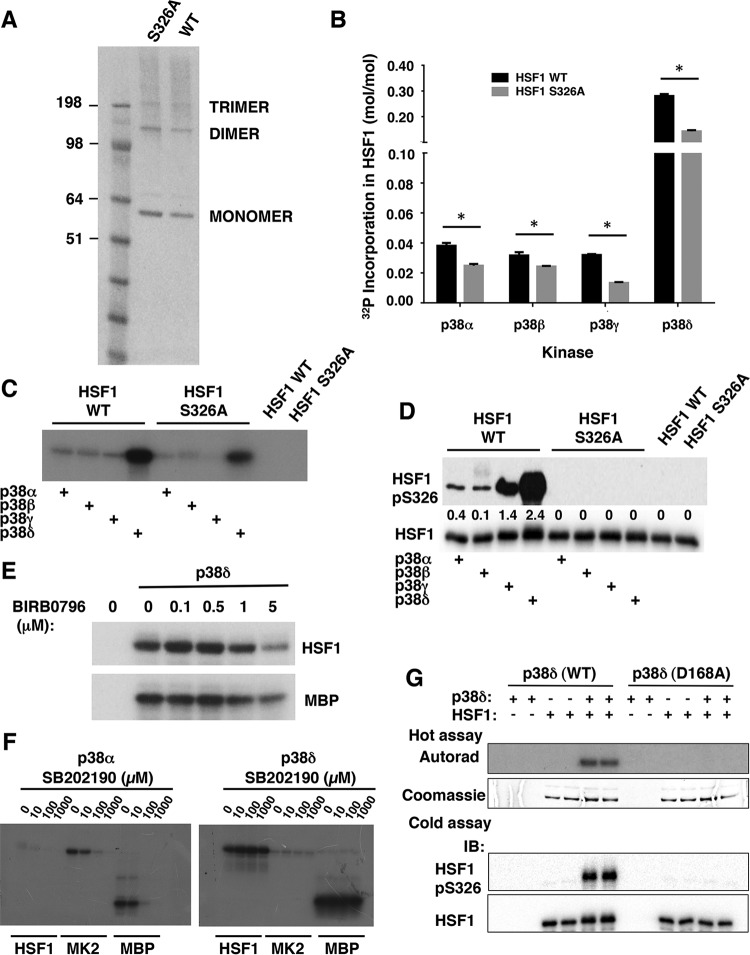
p38 MAPK phosphorylate HSF1 *in vitro*. (A) Electrophoretic mobility (NuPAGE NoVex Bis-Tris 10% gel) of recombinant hexahistidine-tagged HSF1 wild-type (WT) and S326A mutant. (B to G) Purified activated recombinant p38 MAPK isoforms (0.06 mU/μl) were incubated with recombinant wild-type (WT), S326A mutant HSF1, MK2, or myelin basic protein (MBP) (all at 0.1 μg/μl) at 30°C for 15 min in the presence of 10 mM MgCl_2_ and 0.1 mM [γ-^32^P]ATP. Identical reactions were carried out in the presence of increasing concentrations of the p38α/β inhibitor SB202190 or BIRB0796, which inhibits all p38 isoforms. The reactions were terminated by the addition of SDS gel loading buffer, the samples were loaded on SDS-PAGE, and the excess [γ-^32^P]ATP was removed by electrophoresis. (C and E to G) The gels were dried and subjected to autoradiography. After staining with Coomassie brilliant blue, the protein bands were excised and the incorporated radioactivity (B) was determined by scintillation counting. *, *P* < 0.05. (D and F) Purified activated recombinant p38α (0.06 mU/μl) was incubated with recombinant wild-type (WT) or S326A mutant HSF1 (0.1 μg/μl) at 30°C for 15 min in the presence of 10 mM MgCl_2_ and 0.1 mM ATP. The reactions were terminated by the addition of SDS gel loading buffer, the samples were loaded on SDS-PAGE gels, and the phosphorylation of HSF1 at S326 and levels of total HSF1 were detected by Western blotting.

Next, we used recombinant HSF1 to test the ability of purified recombinant p38 MAPK isoforms to phosphorylate HSF1 *in vitro*. HSF1 was expressed as a His-tagged fusion protein in Escherichia coli. Purified His-HSF1 migrated as a major band during NuPAGE at the expected molecular weight and showed a tendency to form spontaneously dimeric and trimeric species (Fig. 6A). The four p38 MAPK isoforms (α, β, γ, and δ) were expressed individually in Escherichia coli from human cDNAs as inactive glutathione *S*-transferase fusion proteins and purified by affinity chromatography on glutathione-Sepharose. Recombinant MKK6 was then used to activate the p38 proteins and subsequently removed by passage through amylose resin. The enzyme activity of each p38 isoform was quantified by the phosphorylation of a standard substrate, myelin basic protein. Each kinase was used at an equivalent specific enzyme activity in reactions with HSF1 as a substrate.

All p38 isoforms were able to rapidly phosphorylate HSF1 (Fig. 6B, black bars). Quantitative analysis of incubation reactions of HSF1 with either p38α, p38β, p38γ, or p38δ and Mg-[γ^32^P]ATP revealed that p38δ phosphorylated HSF1 at a higher rate and stoichiometry than did p38α, p38β, or p38γ, indicating that HSF1 is a better substrate for p38δ than for any of the other p38 isoforms (Fig. 6B [black bars] and C). Parallel reactions in the absence of HSF1 showed that, under these experimental conditions, there was no detectable autophosphorylation of any of the kinases (not shown), and the radioactivity in these samples (as quantified by scintillation counting) was identical to that of the buffer blank. Western blot analysis using the S326-phosphospecific antibody confirmed this conclusion and clearly demonstrated that S326 was one of the phosphorylation sites (Fig. 6D).

The extent of phosphorylation of HSF1 was dependent on the kinase concentration as well as the incubation time. Thus, with 6 mU/μl of enzyme and 60 min of incubation (when phosphate incorporation had reached or was approaching a plateau), the stoichiometry of the reaction was 2, 1.3, 0.8, and 2.2 mol/mol for the α, β, γ, and δ isoforms, respectively (data not shown), suggesting that, under these experimental conditions, p38α, p38β, and p38δ were able to phosphorylate at least two sites on HSF1. With 0.06 mU/μl of enzyme and 15 min of incubation, the stoichiometry of the reaction for HSF1 was 0.04, 0.03, 0.03, and 0.28 mol/mol for p38α, p38β, p38γ, and p38δ, respectively (Fig. 6B, black bars), indicating that p38δ was the most efficient catalyst among the isoforms. The p38δ-mediated phosphorylation of HSF1 was inhibited by BIRB0796 (Fig. 6E), which inhibits all p38 MAPK isoforms (44). In contrast to wild-type (WT) HSF1, under the same experimental conditions, the stoichiometry of the reaction for mutant HSF1, in which S326 was replaced with alanine (S326A) was 0.02, 0.02, 0.01 and 0.14 mol/mol for the p38α, β, γ, and δ isoforms, respectively (Fig. 6B [gray bars] and C), suggesting that S326 is one of the phosphorylation sites and confirming the existence of an additional site(s) on HSF1 which is also phosphorylated by p38 MAPK. Interestingly, this experiment also showed that, although not as robust as p38δ, p38γ was the most selective isoform in phosphorylating S326.

To confirm the unusually high substrate preference of p38δ for HSF1 relative to the more widely studied p38α/β isoforms, we carried out analogous reactions with MK2 as a substrate. In comparison to p38δ, the initial rates of activation of this physiological substrate are ∼20 times faster for the α or β isoforms (47). Indeed, p38δ was far less effective in phosphorylating MK2 than was p38α (Fig. 6F). In sharp contrast, p38δ was more than 20-fold more efficient than p38α in catalyzing the phosphorylation of HSF1. As expected, the p38α-mediated, but not p38δ-mediated, phosphorylation of all three substrates was dose dependently inhibited by the p38α/β inhibitor SB202190.

As mentioned above, we used MBP-MKK6 to obtain active bacterially produced human p38 MAPK. To make absolutely certain that the phosphorylation of HSF1 was not due to any residual MKK6, a new preparation of p38δ was made in parallel: a preparation of a kinase-dead mutant (D168A) version of the enzyme under identical conditions. After incubation and removal of the activating kinase, each protein was used at an equivalent concentration in a reaction with HSF1 as a substrate. Phosphorylation of HSF1 occurred with the active but not the kinase-dead p38δ, as revealed by autoradiography and Western blot analysis (Fig. 6G), establishing the p38δ enzyme as the only HSF1 kinase activity in the preparation.

Together, these data demonstrate that HSF1 can be phosphorylated *in vitro* by all p38 MAPK isoforms at S326 and that p38δ is the most efficient catalyst among the isoforms, whereas p38γ is the most specific. However, it is important to note that S326 is not the only site of phosphorylation by these kinases as the S326A mutant HSF1 was also phosphorylated, albeit with the expected reduction in stoichiometry. To identify the phosphorylation sites precisely, we used a protease-mass spectrometric approach. Recombinant HSF1 was incubated with recombinant p38α or p38δ and, after electrophoretic separation and in-gel proteolytic digestion, the resulting tryptic peptides were analyzed by mass spectrometry. Under these conditions, the sequence coverage was ∼50%. We found that the two p38 isoforms phosphorylated identical sites (Table 1), suggesting that the increased phosphate incorporation with p38δ was due to a higher rate of phosphorylation of the same sites that were phosphorylated by p38α and not due to phosphorylation of an additional site(s). Three phosphorylated peptides were identified in each sample. The corresponding mass (*m/z* 2,902.4659 for p38α and *m/z* 2902.4683 for p38δ) of the longest peptide was in precise agreement with the calculated molecular weight of a peptide containing phosphorylated S326 (*m/z* 2,902.4689, R.VEEASPGRPSSVDTLL**S^326^**PTALIDSILR.E). The mass of the shorter peptides (*m/z* 1,299.5487 and 1,526.7121 for p38α and *m/z* 1,299.5493 and 1,526.7121 for p38δ) corresponded exactly to the molecular weight of peptides K.EEPPSPPQ**S^307^**PR.V (*m/z* 1,299.5496) and R.VKEEPP**S^303^**PPQ**S^307^**PR.V (*m/z* 1,526.7130), in which both S303 and S307 were phosphorylated. Notably, S303 was found in both phosphorylated and unphosphorylated forms. The phosphorylation of HSF1 at S303/307 by the same kinase which phosphorylates the transcription factor at S326 was at first glance surprising, because in contrast to the activating S326 phosphorylation, phosphorylation at S303/307 is inhibitory (14–16). However, this finding provides a possible explanation for the observation that although PEITC treatment causes a concentration-dependent increase in HSF1 phosphorylation (Fig. 3 and 4A), induction of Hsp70 is lower at the higher PEITC concentration (Fig. 1B, 3A, and 4A). Immunoblotting with isoform-specific antibodies showed that p38α, p38γ, and p38δ are well expressed in MDA-MB-231 cells and confirmed that the levels of these kinases did not change upon exposure to PEITC (Fig. 4E).

**TABLE 1:** Phosphopeptides identified by liquid chromatography tandem mass spectrometry^*a*^

Peptide sequence	Phospho-S	*m/z*	
		Calc	Expt
p38 MAPKα + HSF1			
K.EEPP**S^303^**PPQ**S^307^**PR.V	S303/307	1,299.5496	1,299.5487
R.VKEEPP**S^303^**PPQ**S^307^**PR.V	S303/307	1,526.7130	1,526.7121
R.VEEASPGRPSSVDTLL**S^326^**PTALIDSILR.E	S326	2,902.4689	2,902.4659
p38 MAPKδ + HSF1			
K.EEPP**S^303^**PPQ**S^307^**PR.V	S303/307	1,299.5496	1,299.5493
R.VKEEPP**S^303^**PPQ**S^307^**PR.V	S303/307	1,526.7130	1,526.7121
R.VEEASPGRPSSVDTLL**S^326^**PTALIDSILR.E	S326	2,902.4689	2,902.4683

a Phosphopeptides were identified by liquid chromatography-tandem mass spectrometry after 60 min incubation of recombinant human HSF1 (1.0 μg) with p38 MAPKα or p38 MAPKδ (0.06 mU/μl), followed by SDS-PAGE separation and in-gel tryptic digestion. The phosphorylated residue is indicated by boldfacing in column 1. Calc, calculated; Expt, expected.

### Deletion or inhibition of p38γ decreases the phosphorylation of HSF1 at S326 in cells.

**FIG 7:**
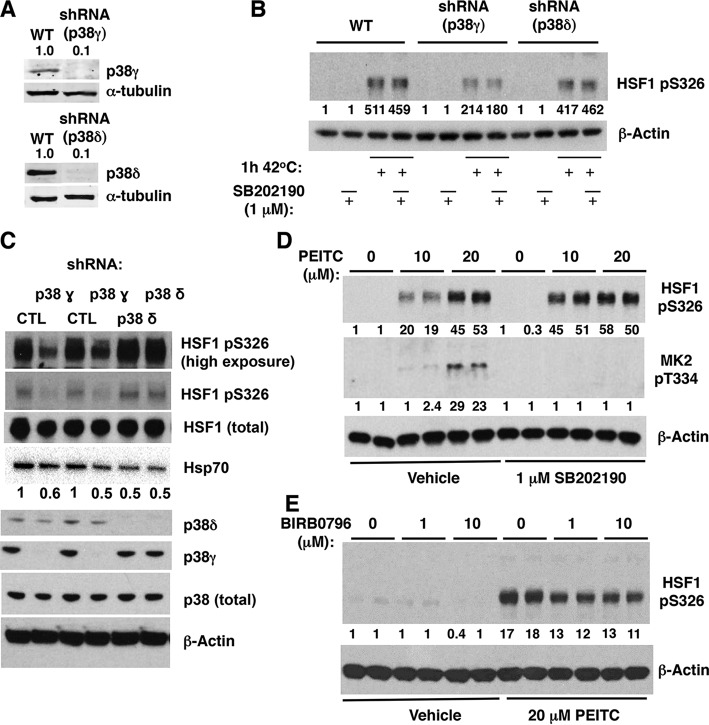
Deletion or inhibition of p38γ MAPK reduces the levels of pS326 HSF1 in cells. (A) Immunoblotting for p38 γ and δ in A431 cells, which either express both p38γ and p38δ (WT) or in which p38γ or p38δ had been stably knocked down using selective shRNA. (B) A431 cells (5 × 10^5^ per well, WT or p38γ or p38δ deficient) were preincubated for 1 h with vehicle (0.1% acetonitrile) or SB202190, and exposed to heat shock (42°C) for a further 1 h. (C) p38γ or p38δ was stably knocked down in MDA-MB-231 cells using selective shRNA. The levels of total HSF1, HSF1 phosphorylated at S326, total p38, p38γ, and p38δ, and Hsp70 were detected by Western blot analysis. (D and E) MDA-MB-231 cells (5 × 10^5^ per well) grown in six-well plates were pretreated with vehicle (0.1% acetonitrile), SB202190, or BIRB0796 for 1 h and subsequently either treated with vehicle (0.1% acetonitrile) or PEITC for a further 1.5 h. HSF1 phosphorylated at S326 (B to E) and MK2 phosphorylated at T334 (D) were detected by Western blot analysis. The levels of α-tubulin (A) or β-actin (B to E) served as loading controls.

To address whether p38γ and p38δ play a role in the phosphorylation of HSF1 at S326 in cells, we first used the human epidermoid cancer cell line A431, in which both p38γ and p38δ are expressed, along with its derivatives in which p38γ or p38δ had been stably knocked down by more than 90%, using selective short hairpin RNA (shRNA) (Fig. 7A) (28). In comparison to the parental cells or cells deficient in p38δ, the heat shock-mediated phosphorylation of HSF1 at S326 was reduced by 60% in cells lacking p38γ (Fig. 7B), in close agreement with the high selectivity of this isoform for the S326 phosphorylation *in vitro* (Fig. 6B to D). Treatment with the p38α/β-specific inhibitor SB202190 had no further effect, indicating that p38α and p38β do not contribute significantly to the phosphorylation of S326 in these cells (Fig. 7B).

Similar results were obtained in MDA-MB-231 cells: shRNA-mediated knockdown (by >90%) of p38γ led to a substantial reduction (by ∼50%) in the phosphorylation of HSF1 at S326 at basal cell culture conditions, whereas the knockdown of p38δ did not have this effect (Fig. 7C). The knockdown of p38γ led to a corresponding decrease in the levels of Hsp70 (Fig. 7C). Interestingly, the levels of Hsp70 were also reduced upon p38δ knockdown, even though the phosphorylation of HSF1 at S326 was not affected. These data suggest that p38δ might be involved in catalyzing the phosphorylation of other (than S326) sites, which activate HSF1, a finding consistent with the highest stoichiometry of the reaction of this p38 isoform *in vitro* with both WT and S326A mutant HSF1 (Fig. 6B and C); the identity of these potential sites is presently unknown.

The conclusion that p38α and p38β do not contribute significantly to the phosphorylation of S326 in A431 cells was further supported by studies in PEITC-treated MDA-MB-231 cells, where SB202190 either had no effect (at the high concentration of PEITC) or even enhanced by 2.5-fold (at the low concentration of PEITC) the levels of pS326 HSF1 after exposure to the isothiocyanate (Fig. 6D). The activation of p38α/β by PEITC and the efficacy of SB202190 were confirmed by monitoring the levels of phosphorylated (at T334) MK2 (Fig. 7D). Finally, we used BIRB0796, which inhibits all four p38 MAPK isoforms (44). Pretreatment with BIRB0796 for 1 h reduced the phosphorylation of S326 in PEITC-treated MDA-MB-231 cells (Fig. 7E). Together, these findings strongly suggest that p38γ is the principal p38 MAPK isoform responsible for the phosphorylation of HSF1 at S326 in cells.

**FIG 8:**
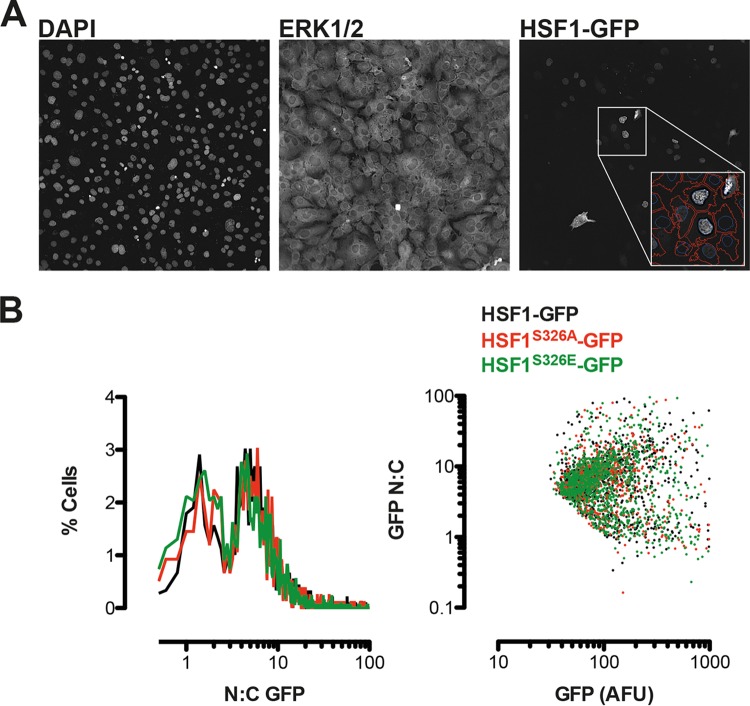
S326A/E mutation does not influence the nucleocytoplasmic distribution of ectopically expressed HSF1-GFP. HSF1-knockout MEFs were transfected with GFP-tagged wild-type or S326A/E mutants of HSF1 prior to counter staining with anti-ERK1/2 antibodies and DAPI. Four fields of view per well of eight replicate wells per condition were imaged using a robotic high-content microscope. Automated and systematic analysis of images was performed using a custom algorithm. (A) A single representative field of view is shown from one well. DAPI and ERK1/2 images were used to define nuclear and cytoplasmic regions, respectively, and GFP fluorescence was recorded from each region (as indicated in the inset screengrab showing automated cell definition), accepting >70 AFU per cell as positively transfected. (B) Plots of single cell data show a frequency histogram of nuclear/cytoplasmic GFP fluorescence (left panel) indicating a bimodal distribution of HSF1, which is unaffected by S326A/E mutation. The right-hand panel shows a comparison of whole-cell GFP fluorescence in the same cell populations versus nucleocytoplasmic distribution, indicating that the bimodal distribution is apparent across a 10-fold difference in HSF1-GFP levels and is again unaffected by S326A/E substitution.

Phosphorylation at S326 but not at any of the other serine residues identified by Guettouche et al. (19) has been shown to affect the heat shock-induced transcriptional activity of HSF1 without affecting the trimerization or nuclear translocation of the transcription factor. In agreement, we found that both purified recombinant wild-type and S326A mutant HSF1 have the propensity to form dimers and trimers spontaneously (Fig. 6A). By use of quantitative high content imaging, we examined the nuclear and cytoplasmic distribution of wild-type or S326A or S326E mutant GFP-HSF1 fusion proteins after their ectopic expression in HSF1-knockout MEFs and did not observe any significant differences among the genotypes (Fig. 8). Notably, however, these results should be interpreted with caution: it is well documented that in C. elegans, ectopic expression of HSF1 produces a gain-of-function phenotype (48, 49), indicating that any level of overexpression of HSF1 may not accurately reflect the physiological properties of the endogenous protein.

## DISCUSSION

By use of mass spectrometry and protein sequencing, Guettouche et al. (19) found that in cells subjected to heat shock, human HSF1 is phosphorylated at 12 serine residues: S121, S230, S292, S303, S307, S314, S319, S326, S344, S363, S419, and S444. More recently, using mass spectrometry-based proteomics, Xu et al. (50) reported the phosphorylation at five additional serine residues (S127, S195, S216, S320, and S368) and at four threonine residues (T142, T323, T367, and T369). The functional significance of most threonine phosphorylations is unknown, except for T142, the phosphorylation of which by casein kinase 2 (CK2) has been reported to increase the transcriptional activity of HSF1 (51). It is well established that phosphorylations at S303/307, S121, and S363 inhibit the function of the transcription factor and are involved in the attenuation phase of the heat shock response (14–16, 18), whereas phosphorylation at S216 by Polo-like kinase 1 (PLK1) promotes the ubiquitination and degradation of HSF1 during mitosis (52). Curiously, PLK1 also phosphorylates HSF1 at S419 but, in contrast to the inhibitory S216 phosphorylation, phosphorylation at S419 is activating and promotes the nuclear translocation of the transcription factor (53). Phosphorylation at S320 by protein kinase A also activates HSF1 (54). Another activating phosphorylation occurs at S230; it is catalyzed by calcium/calmodulin-dependent protein kinase II (CaMKII) and enhances the magnitude of the response upon heat stress (55). Although the DNA-binding ability of the S230A mutant of HSF is retained, its transcriptional activity is reduced by ∼50% in comparison to wild-type HSF1.

Phosphorylation at S326 is a hallmark for HSF1 activation, and several studies have attempted to identify the kinase(s) phosphorylating this site. It was reported that the mechanistic target of rapamycin (mTOR) is able to catalyze the phosphorylation of HSF1 at S326 (20). However, PEITC inhibits mTOR (56). In full agreement, we also found that the mTOR pathway was inhibited by PEITC, as evidenced by the decreased phosphorylation of the ribosomal subunit S6 at S235/236 (Fig. 4B). Overall, our data presented in this contribution imply that mTOR is not the primary kinase responsible for the phosphorylation of S326 on HSF1 in response to treatment with PEITC and further implicate the family of proline-directed p38 MAPK as highly efficient catalysts, which phosphorylate HSF1 rapidly and stoichiometrically. Notably however, neither pharmacological inhibition of p38 MAPK by small molecule inhibitors nor genetic downregulation of p38γ or p38δ eliminated completely the phosphorylation of HSF1 at S326 (Fig. 7). These results imply that although p38γ is the principal isoform within the p38 MAPK family that phosphorylates this site, inactivation of p38γ allows for compensation by other kinases. One such candidate is JNK1/2, which is also activated by PEITC, albeit at a later time point in comparison with p38 MAPK (Fig. 4D). It has been reported that p38 MAPK engage in feedback control loops that suppress the activities of upstream mitogen-activated protein kinase kinase kinases (MAP3Ks), which participate in the activation of JNK, and by disrupting these feedback control loops, inhibition of p38 leads to the hyperactivation of JNK (57). The possibility that JNK1/2 phosphorylates HSF1 at S326 requires further study.

Interestingly and unexpectedly, we found that in addition to S326, p38 MAPK can also catalyze the phosphorylation of HSF1 at S303/307. In contrast to the activating function of the S326 phosphorylation, phosphorylation at S303/307 inhibits the function of HSF1 (14–16). Although surprising, the fact that the same kinase can catalyze phosphorylation of two distinct sites on HSF1 with opposing functional consequences is not unprecedented. As mentioned above, phosphorylation at S216 by PLK1 inhibits HSF1, whereas phosphorylation at S419 by the same kinase activates the transcription factor (52, 53). Our results imply that either p38 MAPK phosphorylate S326 at a higher rate than at S303/307, thus giving a “window” of HSF1 activation due to S326 phosphorylation before the repressive effect of S303/307 phosphorylation takes place or, alternatively, that there is a threshold of p38 MAPK activation below which S326 is the principal target and above which the S303/307 phosphorylation becomes dominant. The second possibility is supported by the fact that induction of Hsp70 is lower upon treatment with the higher (20 μM) compared to the lower (10 μM) concentration of PEITC, whereas the extent of HSF1 phosphorylation is dependent on the dose of PEITC. In addition, the identity of the phosphatases involved may also influence the relative turnover rates of phosphorylation at each site. Dissecting these possibilities requires better tools for quantitation of relative stoichiometry of phosphorylation at S303/307 versus S326 in different cell compartments.

Previous investigations have suggested the possible involvement of MAPK signaling in the activation of HSF1. Thus, loss of the tumor suppressor neurofibromatosis type 1 (NF1) leads to activation of MAPK signaling and HSF1 (6). Chronic exposure of rodent fibroblast cells to heat stress causes phosphorylation of p38 MAPK and induction of Hsp70 (58, 59). In addition, the anti-inflammatory agent sodium salicylate has been reported to activate p38 MAPK, promote HSF1 DNA binding and transcriptional activity, and induce Hsp70 expression (60). Most recently, it was reported that HSF1 physically interacts and is phosphorylated by MEK (45). However, to our knowledge, there are no prior publications linking HSF1 phosphorylation at S326 directly with p38 MAPK activation. In our study, the identification of p38 MAPK as one family of kinases which phosphorylate HSF1 at S326 was greatly facilitated by the observation that PEITC is an exceptionally robust activator of HSE-dependent transcription (Fig. 1F). PEITC shares the ability to induce the heat shock response with celastrol (61, 62), another natural product which, like PEITC, has a characteristic chemical signature, reactivity with sulfhydryl groups (63). Notably, in a screen comprising ∼900,000 small molecules, Calamini et al. (26) discovered new classes of HSF1 activators, all of which, although structurally diverse, bear the same chemical signature. We propose that this chemical property underlies the ability of PEITC to both inhibit Hsp90 and activate p38 MAPK. Finally, pharmacological targeting of p38γ and p38δ has been recently proposed for the treatment of autoimmune and inflammatory diseases, as well as cancer (64). Since HSF1 activation supports malignant transformation (5), this approach holds promise for targeting HSF1 for cancer treatment.
